# Critical appraisal of the adequacy of surgical indications for non-functioning pancreatic neuroendocrine tumours

**DOI:** 10.1093/bjsopen/zrae083

**Published:** 2024-08-06

**Authors:** Stefano Partelli, Anna Battistella, Valentina Andreasi, Francesca Muffatti, Domenico Tamburrino, Nicolò Pecorelli, Stefano Crippa, Gianpaolo Balzano, Massimo Falconi

**Affiliations:** Pancreas Translational and Clinical Research Centre, Pancreatic Surgery Unit, IRCCS San Raffaele Scientific Institute, Milan, Italy; School of Medicine, Vita-Salute San Raffaele University, Milan, Italy; Pancreas Translational and Clinical Research Centre, Pancreatic Surgery Unit, IRCCS San Raffaele Scientific Institute, Milan, Italy; School of Medicine, Vita-Salute San Raffaele University, Milan, Italy; Pancreas Translational and Clinical Research Centre, Pancreatic Surgery Unit, IRCCS San Raffaele Scientific Institute, Milan, Italy; School of Medicine, Vita-Salute San Raffaele University, Milan, Italy; Pancreas Translational and Clinical Research Centre, Pancreatic Surgery Unit, IRCCS San Raffaele Scientific Institute, Milan, Italy; Pancreas Translational and Clinical Research Centre, Pancreatic Surgery Unit, IRCCS San Raffaele Scientific Institute, Milan, Italy; Pancreas Translational and Clinical Research Centre, Pancreatic Surgery Unit, IRCCS San Raffaele Scientific Institute, Milan, Italy; School of Medicine, Vita-Salute San Raffaele University, Milan, Italy; Pancreas Translational and Clinical Research Centre, Pancreatic Surgery Unit, IRCCS San Raffaele Scientific Institute, Milan, Italy; School of Medicine, Vita-Salute San Raffaele University, Milan, Italy; Pancreas Translational and Clinical Research Centre, Pancreatic Surgery Unit, IRCCS San Raffaele Scientific Institute, Milan, Italy; Pancreas Translational and Clinical Research Centre, Pancreatic Surgery Unit, IRCCS San Raffaele Scientific Institute, Milan, Italy; School of Medicine, Vita-Salute San Raffaele University, Milan, Italy

## Abstract

**Background:**

The lack of preoperative prognostic factors to accurately predict tumour aggressiveness in non-functioning pancreatic neuroendocrine tumours may result in inappropriate management decisions. This study aimed to critically evaluate the adequacy of surgical treatment in patients with resectable non-functioning pancreatic neuroendocrine tumours and investigate preoperative features of surgical appropriateness.

**Methods:**

A retrospective study was conducted on patients who underwent curative surgery for non-functioning pancreatic neuroendocrine tumours at San Raffaele Hospital (2002–2022). The appropriateness of surgical treatment was categorized as appropriate, potential overtreatment and potential undertreatment based on histologic features of aggressiveness and disease relapse within 1 year from surgery (early relapse).

**Results:**

A total of 384 patients were included. Among them, 230 (60%) received appropriate surgical treatment, whereas the remaining 154 (40%) underwent potentially inadequate treatment: 129 (34%) experienced potential overtreatment and 25 (6%) received potential undertreatment. The appropriateness of surgical treatment was significantly associated with radiological tumour size (*P* < 0.001), tumour site (*P* = 0.012), surgical technique (*P* < 0.001) and year of surgical resection (*P* < 0.001). Surgery performed before 2015 (OR 2.580, 95% c.i. 1.570 to 4.242; *P* < 0.001), radiological tumour diameter < 25.5 mm (OR 6.566, 95% c.i. 4.010 to 10.751; *P* < 0.001) and pancreatic body/tail localization (OR 1.908, 95% c.i. 1.119 to 3.253; *P* = 0.018) were identified as independent predictors of potential overtreatment. Radiological tumour size was the only independent determinant of potential undertreatment (OR 0.291, 95% c.i. 0.107 to 0.791; *P* = 0.016). Patients subjected to potential undertreatment exhibited significantly poorer disease-free survival (*P* < 0.001), overall survival (*P* < 0.001) and disease-specific survival (*P* < 0.001).

**Conclusions:**

Potential overtreatment occurs in nearly one-third of patients undergoing surgery for non-functioning pancreatic neuroendocrine tumours. Tumour diameter emerges as the sole variable capable of predicting the risk of both potential surgical overtreatment and undertreatment.

## Teaser

Treatment appropriateness was investigated for patients undergoing surgery for resectable non-functioning pancreatic neuroendocrine tumours. Of note, potential overtreatment occurred in nearly one-third of the treated patients and tumour diameter emerged as the sole variable capable of predicting both potential overtreatment and undertreatment. Although an increase in the percentage of appropriately treated patients has been observed over the past decade, an accurate assessment of lesions with a small radiological diameter could lead to further improvements in the selection of surgical treatment.

## Introduction

Non-functioning pancreatic neuroendocrine tumours (NF-PanNETs) have historically been considered rare neoplasms, but their incidence has significantly risen over the past two decades due to the widespread use of high-quality imaging techniques^1^. NF-PanNETs encompass a heterogenous group of lesions with varying biological behaviours, ranging from indolent to highly aggressive tumours^2^. Risk stratification of NF-PanNETs heavily relies on postoperative histopathological features. Tumour stage, grade, necrosis, perineural and microvascular invasion have emerged as the most relevant prognostic factors in patients undergoing surgery for NF-PanNETs^3–6^. However, the availability of preoperative predictors of aggressiveness remains limited^7–11^. Consequently, achieving appropriate and tailored management of these neoplasms poses a significant challenge.

Surgery represents the mainstay of curative treatment for localized NF-PanNETs^12,13^. Despite the high curative rates associated with surgical resection, approximately 15–30% of patients experience disease recurrence within 5 years from surgery^14–17^. Given these findings, surgeons must carefully evaluate the risks associated with treatment decisions. In certain patients, surgery may represent a futile intervention, prompting consideration of multimodal treatment approaches to minimize the risk of early recurrence. Conversely, in other cases, a non-operative treatment may be a more appropriate therapeutic choice, particularly for patients with lesions before surgery deemed to have a low risk of aggressiveness. Indeed, recent studies^18–20^ have evaluated and confirmed the safety of ‘active surveillance’ as an alternative to surgical resection for patients affected by sporadic, small, asymptomatic NF-PanNETs. Thus, in the presence of specific clinico-radiological features, surgery may constitute overtreatment, subjecting patients to an unnecessary and potentially harmful procedure. Notably, pancreatic surgery carries a risk of high perioperative morbidity rate, including major complications^21,22^ as well as long-term pancreatic functional impairment^23^.

The aims of this study were to: i) evaluate the appropriateness of surgical treatment in patients undergoing surgery for NF-PanNETs and ii) investigate the preoperative features predicting the likelihood of exposing the patients to potential overtreatment or potential undertreatment in this setting.

## Methods

### Study design

The present retrospective observational study adhered to the STROBE guidelines^24^. All consecutive patients who underwent potentially curative surgery (R0–R1) for NF-PanNETs at San Raffaele Hospital (Milan, Italy) from November 2002 to March 2022 were considered. Exclusion criteria encompassed patients under the age of 18 years, patients with functioning neoplasms and individuals diagnosed with poorly differentiated pancreatic neuroendocrine carcinomas (PanNECs). Palliative tumour resection (R2) cases were also excluded. The flow of patients from initial screening to the final study sample is depicted in *Fig. S1*. Given the retrospective nature of the study, ethical committee approval was not required.

### Definition of surgical treatment appropriateness

The appropriateness of surgical treatment was categorized into three groups: potential overtreatment, appropriate treatment and potential undertreatment, based on final histologic findings and occurrence of disease relapse within 1 year following surgery.

Radical resection (R0/R1 resection) was achieved in all patients across the three categories, including in the presence of distant metastases.

The potential overtreatment group included patients who underwent radical surgical resection but had no histologic evidence of tumour aggressiveness (that is G1, T1–T2, N0, M0, no microvascular and/or perineural invasion) and did not experience disease recurrence.

The appropriate surgical treatment group included patients who underwent radical surgical resection and had histologic evidence of at least one feature of aggressiveness (that is G2–G3, T3–T4, N1, M1, microvascular or perineural invasion) but did not experience disease recurrence within 1 year following surgery.

The potential undertreatment group comprised patients who underwent radical surgical resection and experienced disease recurrence within 1 year from surgery.

### Data collection

A comprehensive collection of preoperative, intraoperative and postoperative data was conducted by retrospectively retrieving information from a prospectively maintained institutional database. Preoperative variables, including demographic characteristics (age and sex), body mass index (BMI) and presenting symptoms were reviewed. Performed diagnostic procedures, including computed tomography (CT), magnetic resonance imaging (MRI), endoscopic ultrasound (EUS) and ^68^Gallium positron emission tomography (^68^Ga-PET), were documented, along with the radiological tumour site (head/uncinate process *versus* body/tail) and size (maximum diameter assessed by imaging techniques). The date of surgery was categorized into four surgical time intervals (2002–2007, 2008–2012, 2013–2017, 2018–2022) for analysis. Intraoperative parameters included the type of surgery (pancreatoduodenectomy, distal pancreatectomy, total pancreatectomy, enucleation, middle pancreatectomy), surgical approach (laparoscopic *versus* open) and vascular resection. The duration of hospital stay (LOS) was calculated from the date of surgery to the date of discharge. Postoperative complications were classified according to the Clavien–Dindo classification of surgical complications^25^. Postoperative pancreatic fistula (POPF) was graded according to the 2016 definition proposed by the International Study Group on Pancreatic Surgery (ISGPS)^26^. Long-term pancreatic impairment (endocrine and/or exocrine insufficiency) occurring after surgery was also recorded. Tumour grade was determined based on the 2017 World Health Organization (WHO) classification into G1 (Ki67 < 3%), G2 (Ki67 3–20%) and G3 (Ki67 > 20%)^27^. The Ki67 proliferative index was assessed by MIB1 antibody staining and expressed as the percentage of cells with nuclear staining in 2000 cells, counted in the area of highest nuclear labelling^28^. Tumour stage was categorized following the current European Neuroendocrine Tumour Society (ENETS) TNM staging system^29^. The status of the surgical margins was evaluated and classified as R0 (no residual tumour) and R1 (microscopic residual tumour). R1 resection was defined as the presence of microscopic residual tumour at the resection margins, or in the presence of a minimum margin length ≤1 mm. Additionally, the presence of microvascular invasion, perineural invasion and necrosis was reviewed.

### Definition of survival outcomes

Disease-free survival (DFS) was defined as the time from surgery to any kind of disease recurrence. Overall survival (OS) was calculated as the time from surgery to the date of death for any cause, and censored. Disease-specific survival (DSS) was defined as the time from surgery to disease-related death. DFS, OS and DSS were censored at the last follow-up.

### Statistical analysis

Categorical variables were presented as absolute numbers with corresponding percentages, and compared using the χ^2^ or Fisher’s exact test, as appropriate. Continuous variables were reported as median with interquartile ranges (i.q.r.) for skewed distribution, or as mean (s.d.) for normal distributions. The normality of continuous variables was assessed using the Kolmogorov–Smirnov test. Continuous variables were compared between two groups by using the Student’s *t* test or Mann–Whitney *U* test, as appropriate based on the distribution of the variables. The Kruskal–Wallis test was performed to compare continuous variables between multiple groups. Bonferroni correction was applied to account for multiple comparisons, both for categorical and continuous variables. Receiver operating characteristic (ROC) curve analysis was performed to evaluate radiological tumour size as a predictor of treatment appropriateness and to find the best cut-off to identify patients at higher risk of overtreatment before surgery. The global performance was expressed as area under the curve (AUC). Multivariable logistic regression analysis was performed to identify preoperative predictors of potential surgical overtreatment and undertreatment. Only variables that exhibited significant associations with treatment appropriateness in the univariate analysis were included in the initial multivariable model. A backward stepwise selection procedure was employed to derive the final multivariable model. When necessary, continuous variables were categorized based on their median value.

Survival probability was estimated using the Kaplan–Meier method. The log-rank test was employed to compare DFS, OS and DSS among the potential overtreatment, potential undertreatment and appropriate treatment groups. Statistical significance was set at a *P* value less than 0.05. All statistical analyses were performed using SPSS version 26.0 for Mac software (SPSS, Inc., Chicago, IL, USA).

## Results

### Study participants

**Fig. 1:**
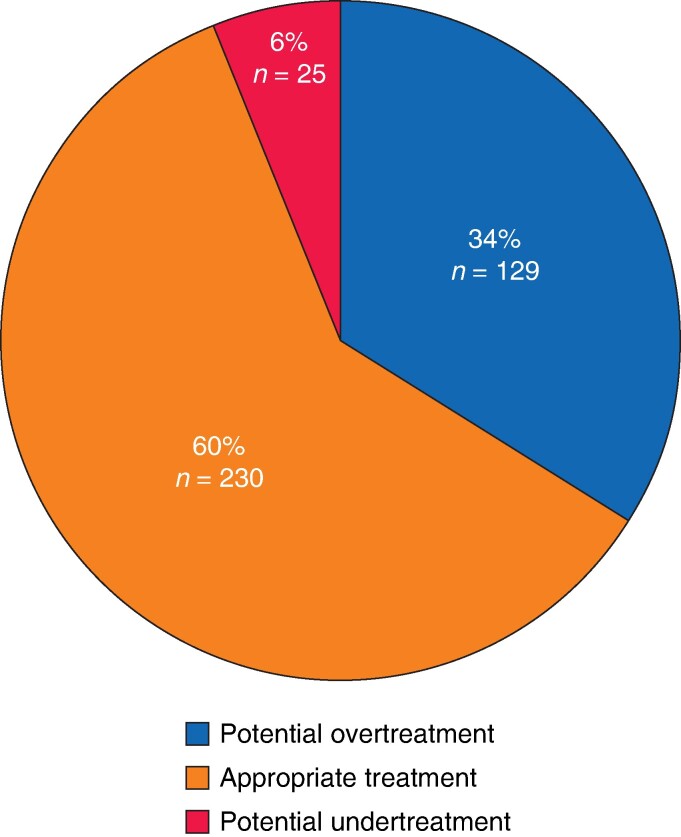
Patients who underwent surgery for non-functioning pancreatic neuroendocrine tumours (NF-PanNETs) and received potential overtreatment, appropriate treatment or potential undertreatment

A total of 384 patients who underwent surgery for resectable NF-PanNETs were included in the study. Among them, 230 (60%) received appropriate surgical treatment, whereas 129 (34%) experienced potential overtreatment and 25 (6%) were potentially undertreated (*Fig. 1*).

### Demographic and clinico-radiological features

Demographics and clinico-radiological characteristics were compared among the three groups, as presented in *Table 1*. The analysis revealed a significant association between the type of diagnostic work-up and the appropriateness of surgical treatment (*P* = 0.034). Among the patients within the appropriate surgical treatment group (*n* = 230), the most common diagnostic work-up consisted of a combination of high-quality imaging (CT and/or MRI), EUS and ^68^Gallium PET (^68^Ga PET), performed in 123 patients (54%). A significant difference in the type of diagnostic work-up employed was observed during the study interval (*P* < 0.001). Specifically, over the past decade, assessment of disease extension has become more accurate and comprehensive through the use of multiple imaging modalities (CT and/or MRI + EUS + ^68^Ga PET) (2002–2007: 2 of 27, 7%; 2008–2012: 13 of 118, 11%; 2013–2017: 69 of 121, 57%; 2018–2022: 94 of 118, 80%) (*Fig. S2*). Moreover, a significant increase in the interval from diagnosis to surgical resection was observed during the study interval (2002–2007: 1 month (i.q.r. 0–2), 2008–2012: 1 month (i.q.r. 1–2.25), 2013–2017: 3 months (i.q.r. 2–7), 2018–2022: 3 months (i.q.r. 2–7.75); *P* < 0.001).

**Table 1:** Comparison of demographics, clinical and preoperative characteristics between patients who underwent surgery for non-functioning pancreatic neuroendocrine tumours (NF-PanNETs) and received potential overtreatment (*n* = 129), appropriate treatment (*n* = 230) and potential undertreatment (*n* = 25)

Variable	Potential overtreatment *n* = 129	Appropriate treatment *n* = 230	Potential undertreatment *n* = 25	Overall P*	Adj. *P*† PO *versus* AT	Adj. *P*‡ PO *versus* PU	Adj. *P*§ AT *versus* PU
**Sex**				0.487	1.000	0.834	0.696
Male	72 (56)	130 (56)	11 (44)				
Female	57 (44)	100 (44)	14 (56)				
Age at surgery (years), median (i.q.r.)	60 (48–67)	60 (49–68)	64.5 (48.5–73.7)	0.793	1.000	1.000	1.000
BMI (kg/m^2^), median (i.q.r.)	25.1 (22.8–28.7)	24.82 (22.8–28.3)	22.4 (19.8–25.8)	0.155	1.000	0.116	0.245
Symptoms at diagnosis	16 (12)	45 (20)	4 (16)	0.235	0.255	1.000	1.000
**Number of imaging examinations**				0.068	0.033	1.000	1.000
One imaging modality	28 (22)	37 (16)	4 (16)				
Two imaging modalities	42 (33)	48 (21)	6 (24)				
Three imaging modalities	34 (26)	73 (32)	9 (36)				
Four imaging modalities	25 (19)	72 (31)	6 (24)				
**Diagnostic work-up**				**0**.**034**	**0**.**021**	1.00	1.00
CT or MRI	28 (22)	37 (16)	4 (16)			0	0
CT and MRI	4 (3)	5 (2)	0 (0)				
CT and/or MRI + EUS or ^68^Ga PET	52 (40)	65 (28)	11 (44)				
CT and/or MRI + EUS + ^68^Ga PET	45 (35)	123 (54)	10 (40)				
Radiological diameter (mm), median (i.q.r.)	20 (15–27)	30.5 (24–47.25)	34 (30–63.75)	**<0**.**001**	**<0**.**001**	**<0**.**001**	0.833
**Tumour site**				**0**.**012**	**0**.**009**	0.63	1.00
Head/uncinate process	31 (24)	91 (40)	9 (36)			6	0
Body/tail	98 (76)	139 (60)	16 (64)				

Values are *n* (%) unless otherwise indicated. Values in bold indicate statistical significance. BMI, body mass index; CT, computed tomography; MRI, magnetic resonance; PET, positron emission tomography; EUS, endoscopic ultrasound; Adj, adjusted; PO, potential overtreatment; AT, appropriate treatment; PU, potential undertreatment.

^*^Potential overtreatment *versus* appropriate treatment *versus* potential undertreatment.

^†^Potential overtreatment *versus* appropriate treatment, *P* values with Bonferroni correction for multiple comparisons.

^‡^Potential overtreatment *versus* potential undertreatment, *P* values with Bonferroni correction for multiple comparisons.

^§^Appropriate treatment *versus* potential undertreatment, *P* values with Bonferroni correction for multiple comparisons.

An increase in the rate of appropriately treated patients has been observed after the introduction of institutional multidisciplinary meetings (2018), with a concurrent decrease in potentially overtreated patients (appropriate treatment: 90 of 119, 76% *versus* 140 of 265, 53%, potential overtreatment: 21 of 119, 7% *versus* 108 of 265, 41%; *P* < 0.001). No significant changes in the rate of potentially undertreated patients were reported (8 of 119, 7% *versus* 17 of 265, 6%).

Patients with tumours located in the pancreatic body-tail exhibited a significantly higher frequency of potential overtreatment (39%, 98 of 253) compared with patients with pancreatic head lesions (31 of 131, 24%; *P* = 0.012). Furthermore, a smaller radiological diameter was significantly associated with potential overtreatment (potential overtreatment: 20 mm (i.q.r. 15–27), appropriate treatment: 30 mm (i.q.r. 24–47), potential undertreatment: 34 mm (i.q.r. 30–64); *P* < 0.001). By ROC curve analysis (*Fig. S3*), a radiological tumour size of 25.5 mm was identified as the most accurate cut-off, demonstrating 71% sensitivity and 74% specificity in predicting potential overtreatment. The global performance of tumour radiological diameter as a predictor of potential overtreament was deemed adequate (AUC 0.783, 95% c.i. 0.736 to 0.830, *P* < 0.001).

### Intraoperative features and surgical outcomes

**Fig. 2:**
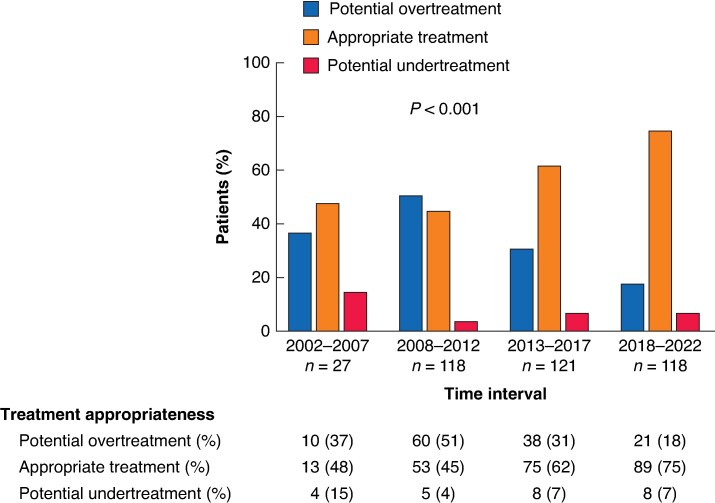
Comparison of rates of potential overtreatment, appropriate treatment and potential undertreatment between patients who underwent surgery for non-functioning pancreatic neuroendocrine tumours (NF-PanNETs) over the study interval (2002–2022), categorized into four subintervals

A comparison of intraoperative features and surgical outcomes among the three groups is reported in *Table 2*. The year of surgical resection demonstrated a strong correlation with treatment appropriateness (*P* < 0.001), revealing a significant increase in the rate of appropriately surgically treated patients during the study interval (2002–2007: 48%, 2008–2012: 45%, 2013–2017: 62%, 2018–2022: 75%). Simultaneously, a concurrent decrease in the rate of potentially overtreated patients was reported (2002–2007: 37%, 2008–2012: 51%, 2013–2017: 31%, 2018–2022: 18%) (*Fig. 2*).

**Table 2:** Comparison of intraoperative characteristics and postoperative outcomes between patients who underwent surgery for non-functioning pancreatic neuroendocrine tumours (NF-PanNETs) and received potential overtreatment (*n* = 129), appropriate treatment (*n* = 230) and potential undertreatment (*n* = 25)

Variable	Potential overtreatment *n* = 129	Appropriate treatment *n* = 230	Potential undertreatment *n* = 25	Overall P*	Adj. *P*† PO *versus* AT	Adj. *P*‡ PO *versus* PU	Adj. *P*§ AT *versus* PU
**Date of surgery**				**<0.001**	**<0.001**	0.102	0.867
2002–2007	10 (8)	13 (6)	4 (16)				
2008–2012	60 (46)	53 (23)	5 (20)				
2013–2017	38 (29)	75 (32)	8 (32)				
2018–2022	21 (16)	89 (39)	8 (32)				
**Type of surgery**				**<0.001**	**<0.001**	0.111	1.000
Pancreatoduodenectomy	18 (14)	84 (37)	8 (32)				
Distal pancreatectomy	74 (57)	127 (55)	15 (60)				
Total pancreatectomy	1 (1)	7 (3)	1 (4)				
Enucleation	30 (23)	10 (4)	1 (4)				
Middle pancreatectomy	6 (5)	2 (1)	0 (0)				
**Parenchyma-sparing resection**				**<0.001**	**<0.001**	**0.039**	1.000
No	93 (72)	218 (95)	24 (96)				
Yes	36 (28)	12 (5)	1 (4)				
**Surgical approach**				0.209	1.000	0.237	0.396
Laparoscopic	45 (35)	72 (31)	5 (20)				
Open	84 (65)	158 (69)	20 (80)				
Vascular resection	0 (0)	12 (5)	4 (16)	**<0.001**	**0.015**	**0.003**	0.174
**Status of resection margins**				**0.002**	0.126	**0.003**	0.087
R0	125 (97)	210 (91)	19 (76)				
R1	4 (3)	20 (9)	6 (24)				
Duration of hospital stay (days), median (i.q.r.)¶,#	9 (6–11)	9 (7–12)	9 (7–11)	0.770	1.000	1.000	1.000
**Postoperative complications^25^**				0.265	0.753	0.462	1.000
No	36 (28)	66 (29)	10 (40)				
Clavien–Dindo I–II	77 (60)	121 (53)	10 (40)				
Clavien–Dindo III–IV–V	16 (12)	43 (19)	5 (20)				
**CR-POPF** ^26^				0.088	0.459	1.000	0.192
No	106 (82)	174 (76)	23 (92)				
Yes							
Grade B	21 (16)	53 (23)	1 (4)				
Grade C	2 (2)	3 (1)	1 (4)				
Exocrine insufficiency	34 (26)	89 (39)	14 (56)	**0.006**	0.054	**0.009**	0.282
Endocrine insufficiency	36 (28)	54 (23)	5 (20)	0.551	1.000	1.000	1.000

Values are *n* (%) unless otherwise indicated. Values in bold indicate statistical significance. Adj, adjusted; CR-POPF, clinically relevant postoperative pancreatic fistula; PO, potential overtreatment; AT, appropriate treatment; PU, potential undertreatment.

^*^Potential overtreatment *versus* appropriate treatment *versus* potential undertreatment. †Potential overtreatment *versus* appropriate treatment, *P* values with Bonferroni correction for multiple comparisons.

^‡^Potential overtreatment *versus* potential undertreatment, *P* values with Bonferroni correction for multiple comparisons.

^§^Appropriate treatment *versus* potential undertreatment, *P* values with Bonferroni correction for multiple comparisons.

^¶^Expressed as median (i.q.r.).

^#^Missing data *n* = 1 potential overtreatment, *n* = 2 appropriate treatment, *n* = 1 potential undertreatment.

The type of surgical intervention was significantly associated with treatment appropriateness (*P* < 0.001).

Parenchyma-sparing resections were more common in patients who received potential overtreatment (28%, *n* = 36) compared with patients who experienced appropriate treatment (5%, *n* = 12; *P* < 0.001) or potential undertreatment (4%, *n* = 1; *P* = 0.039). Pancreatoduodenectomy was more frequently performed in the appropriate treatment group (37%, *n* = 84). Patients receiving potential undertreatment underwent vascular resection in 16% of cases (4 of 25), a percentage significantly higher than the one observed in the potentially overtreated patients (0%; *P =* 0.003). Interestingly, among patients undergoing pancreatic resection with vascular reconstruction, 75% (12 of 16) received an appropriate treatment. However, no significant difference was observed between patients within the potential undertreatment group (5%, 12 of 130) and those appropriately treated (*P* = 0.174).

R1 resections were more frequent (24%) in the potential undertreatment group. The potential overtreatment group had a significantly higher rate of R0 resections compared with the potential undertreatment one (potential overtreatment: 97%, *n* = 125 *versus* potential undertreatment: 76%, *n* = 19; *P* = 0.003).

Following surgical resection, the rate of pancreatic exocrine insufficiency progressively increased across the potential overtreatment (26%), appropriate treatment (39%) and potential undertreatment (56%) groups (*P* = 0.006). The frequency of pancreatic endocrine insufficiency was similar among the three categories (potential overtreatment: 28%, appropriate treatment: 23%, potential undertreatment: 20%; *P* = 0.551).

### Pathological findings

A comparison of pathological features between patients receiving appropriate surgery and potential undertreatment is depicted in *Table S1*. Potentially overtreated patients were excluded from this analysis as they showed no signs of aggressiveness by definition. The median Ki67 proliferative index was significantly higher in patients who experienced potential undertreatment compared with individuals receiving appropriate treatment (8 (i.q.r. 5–18) *versus* 3 (i.q.r. 2–6); *P* < 0.001). Consistently, a significantly higher percentage of G3 tumours was reported in the potential undertreatment group (16% *versus* 2%, *P* < 0.001). Patients in the appropriate treatment group had a significantly lower frequency of T3–T4 tumours (64% *versus* 35%, *P* = 0.005), nodal metastases (76% *versus* 46%, *P* = 0.004), distant metastases (24% *versus* 6%, *P* = 0.007), microvascular invasion (88% *versus* 54%, *P* = 0.001), perineural invasion (52% *versus* 32%, *P* = 0.042) and necrosis (36% *versus* 9%; *P* = 0.001) compared with potentially undertreated subjects. Among the appropriately treated patients with T3–T4 tumours (81 of 230), 54% had a G2–3 PanNET (44 of 81), 51% showed nodal involvement at histological examination (41 of 81), and 28% were positive for both features aggressiveness (G2–3, N1; 23 of 81). Regarding the potential undertreatment group, G2–3 lesions and lymph node metastases were reported in 75% (*n* = 12 of 16) and 69% (11 of 16) of patients with T3–4 tumours respectively. Both characteristics were observed in 56% of patients (9 of 16).

### Preoperative determinants of potential overtreatment and potential undertreatment

Multivariable regression analyses assessing preoperative predictors of potential surgical overtreatment and undertreatment are reported in *Table 3*. Surgical resection performed before 2015 (OR 2.580, 95% c.i. 1.570 to 4.242; *P* < 0.001), radiological tumour size <25.5 mm (OR 6.566, 95% c.i. 4.010 to 10.751; *P* < 0.001) and tumour location in the pancreatic body/tail (OR 1.908, 95% c.i. 1.119 to 3.253; *P* = 0.018) were identified as independent predictors of potential overtreatment. Radiological tumour size was the only independent determinant of potential undertreatment (OR 0.291, 95% c.i. 0.107 to 0.791; *P* = 0.016).

**Table 3:** Multivariate analysis of predictors of potential overtreatment and potential undertreatment between patients who underwent surgery for non-functioning pancreatic neuroendocrine tumours (NF-PanNETs)

Variable	Potential overtreatment		Potential undertreatment	
	OR (95% c.i.)	*P*	OR (95% c.i.)	*P*
**Radiological tumour size**		**<0.001**		**0.016**
≥25.5 mm	1		1	
<25.5 mm	6.566 (4.010, 10.751)		0.291 (0.107, 0.791)	
**Diagnostic work-up**		0.443		0.417
CT and/or MRI	1		1	
CT and/or MRI + EUS and/or ^68^Ga PET	0.787 (0.426, 1.452)		1.604 (0.513, 5.011)	
**Time of surgery**		**<0.001**		0.476
2016–2022	1		1	
2002–2015	2.580 (1.570, 4.242)		0.739 (0.322, 1.697)	
**Lesion site**		**0.018**		0.924
Head/uncinate process	1		1	
Body/tail	1.908 (1.119, 3.253)		0.959 (0.406, 2.268)	

Values in bold indicate statistical significance. OR, odds ratio; CT, computed tomography; MRI, magnetic resonance; PET, positron emission tomography; EUS, endoscopic ultrasound.

### Survival analysis

After a median follow-up of 61 months (i.q.r. 55–66 months), 67 patients (17%) experienced disease recurrence and 29 (7.5%) eventually died due to any cause. Disease relapse was observed in 100% (25 of 25), 19% (42 of 230) and 0% (0 of 129) of patients receiving potential undertreatment, appropriate treatment and potential overtreatment respectively (*P* < 0.001). Potentially overtreated patients showed a 3-year DFS rate of 100% compared with 87 and 0% in patients within the appropriate treatment and potential undertreatment groups respectively (*Fig. S4A*). No significant difference was observed in the site of disease recurrence between patients who received potential undertreatment compared with appropriately treated ones (*P* = 0.225). Among the 42 appropriately treated patients who experienced disease recurrence, 17% (*n =* 7) had a nodal relapse and 83% (*n =* 35) developed distant metastases. Similarly, of the 25 patients who received potential undertreatment, 2 (8%) experienced local recurrence, 5 (20%) developed nodal metastases and 18 (72%) distant metastases.

Patients who received potential undertreatment had the poorest OS (3-year OS 70%) compared with appropriately treated (3-year OS 97%) and potentially overtreated patients (3-year OS 99%) (*P* < 0.001). Overall, 14 patients died of disease. No disease-related deaths occurred in the potential overtreatment group. Patients of the potential undertreatment group also had a worse DSS (3-year DSS 70%) compared with the other groups (3-year DSS 100% for both) (*P* < 0.001) (*Fig. S4B*). No statistically significant differences in terms of OS and DSS were reported between potentially overtreated and appropriately treated patients (OS, *P* = 0.129; DSS, *P* = 0.069).

## Discussion

This study investigated the appropriateness of surgical treatment and associated factors in patients with resectable NF-PanNETs. Surgery for NF-PanNETs presents numerous challenges due to the unique biological behaviour of these tumours. The inherent heterogeneity in aggressiveness poses a significant risk of inadequate surgical resection, resulting in both potential overtreatment and futile interventions.

The current study investigated the suitability of surgical management in a large, single-institution series of 384 patients submitted to curative surgery for NF-PanNET at a tertiary referral centre. The decision to proceed with surgery was found to be appropriate in the majority of patients (60%), nevertheless a significant proportion of cases of potential overtreatment and of futile resection was identified. The rate of potentially overtreated cases decreased over time, which may be attributed to the evolution in the management of small, asymptomatic NF-PanNETs as well as to the advancement in imaging techniques allowing enhanced characterization and identification of these lesions. Indeed, recent studies have shown the feasibility and safety of a ‘watch and wait’ approach for asymptomatic sporadic NF-PanNETs ≤ 2 cm^18,19,30^, leading to a reduction in the risk of overtreatment. Moreover, the current study revealed that patients who underwent surgical resection before 2015 were more likely to experience potential overtreatment. This finding can be attributed to the publication of the ENETS^13^ consensus guidelines in 2016, which recommended conservative management instead of a surgical approach for this particular subset of patients. These guidelines likely influenced clinical practice and led to a shift in the management of small, asymptomatic NF-PanNETs. Furthermore, the introduction of institutional multidisciplinary meetings in 2018 has likely contributed to the increase in the rate of patients receiving appropriate treatment, ensuring a comprehensive assessment of patients’ management. Nevertheless, the rate of cases receiving potential undertreatment remained stable over the study interval, indicating that there have been no significant advances in the management of localized aggressive lesions in the past 20 years. Patients experiencing early disease relapse after surgery might benefit from multimodal treatment approaches. A previous small series reported promising results in this setting, indicating improved oncological outcomes when patients underwent sequential treatments such as surgery preceded by peptide receptor radionuclide therapy^31–33^. However, larger and more rigorous studies are needed to validate the effectiveness of perioperative medical treatments in this specific context.

The findings of the current study, which identified small tumour diameter and pancreatic body/tail location as independent predictors of potential overtreatment, as well as radiological tumour size as a preoperative determinant of potential undertreatment, are consistent with several prior reports in the literature^3–6^. Previous studies^18,20^ have highlighted the challenge of appropriately managing small tumours, particularly those located in the pancreatic body/tail. Importantly, the current study identified a diameter of 25.5 mm as the optimal cut-off for preoperative assessment, effectively distinguishing patients at high risk of overtreatment. This finding implies that patients with tumours smaller than 25.5 mm should undergo careful evaluation before making treatment decisions. Indeed, such patients could be suitable candidates for active surveillance or potentially benefit from a parenchyma-sparing resection approach.

Furthermore, it is reasonable to speculate that the observed correlation between tumour location and potential overtreatment may be attributed to the fact that surgical resections for tumours situated in the pancreatic body/tail (that is distal pancreatectomy) are comparatively less technically challenging and associated with lower postoperative morbidity rates, in contrast to resections required for pancreatic head lesions (that is pancreatoduodenectomy)^34^. Consequently, surgeons may be more inclined to opt for surgical intervention in cases involving pancreatic body-tail lesions, even in the absence of clear preoperative indications of aggressiveness.

Another notable finding from this study pertains to the detrimental consequences of potential unnecessary surgical interventions. Noteworthy is the rate of postsurgical morbidity among potentially overtreated patients, which has significant implications. Alarmingly, the current results indicate that 25% of potentially overtreated patients experienced postoperative pancreatic impairment, consequently impairing their quality of life^23,35,36^. In addition, potentially undertreated patients showed lower overall survival compared with those reported in the literature for patients undergoing curative surgery for locally advanced^37^ and metastatic^38,39^ PanNETs.

The current study has several limitations that should be recognized. The first one is related to its retrospective design. Second, a referral bias might be present, as only patients submitted to surgery in a tertiary centre were considered in this series. Moreover, surgical outcomes were not compared with those of non-operative control groups, which does not allow for the validation of the patients’ classification. In addition, variables such as tumour growth and patients’ choice could not be included in the analysis, thereby precluding an assessment of their impact on surgical indications. Finally, the study developed over a long interval, during which significant evolutions in PanNETs assessment and management as well as advances in surgical techniques occurred.

In conclusion, the study provides insights into the treatment appropriateness for surgically managed NF-PanNETs over a 20-year interval. Potential overtreatment remains a concern, but the rate of appropriately treated patients has been increasing. Surgeons could enhance their clinical judgment and tailor treatment approaches to improve treatment selection, especially in the presence of tumours located in the pancreatic body-tail and/or with a radiological diameter < 25.5 mm. Further research is needed to increase current ability to predict tumour aggressiveness before surgery.

## Supplementary Material

zrae083_Supplementary_Data

## Data Availability

The data that support the findings of this study are available from the corresponding author (M.F.) upon reasonable request.
