# Supplementary material for: Critical appraisal of the adequacy of surgical indications for non-functioning pancreatic neuroendocrine tumours
Source: BJS Open. 2024 Aug 6;8(4):zrae083. doi: 10.1093/bjsopen/zrae083 (PMC11303005; doi:10.1093/bjsopen/zrae083)
Supplement: zrae083_Supplementary_Data [file zrae083_supplementary_data.zip › supplementary_material.docx]

**A critical appraisal of the adequacy of surgical indications for Nonfunctioning Pancreatic Neuroendocrine Tumours**

Stefano Partelli^1,2^^, Anna Battistella^1,2^^, Valentina Andreasi^1,2^, Francesca Muffatti^1^, Domenico Tamburrino^1^, Nicolò Pecorelli^1,2^, Stefano Crippa^1,2^, Gianpaolo Balzano^1^, Massimo Falconi^1,2^

^1^ Pancreas Translational and Clinical Research Centre, Pancreatic Surgery Unit, IRCCS San Raffaele Scientific Institute, Milan, Italy

^2^ Vita-Salute San Raffaele University, Milan, Italy

^These Authors share the first authorship

**Corresponding Author:**

Massimo Falconi, MD
Pancreas Translational and Clinical Research Center, Pancreatic Surgery Unit, IRCCS San Raffaele Scientific Institute, Milan, Italy. School of Medicine, Vita-Salute University, Milan, Italy
Via Olgettina, 60 20132, Milan, Italy
Phone: +39 0226436046 Fax: + 39 0226437807
Email: falconi.massimo@hsr.it
ORCID ID: 0000-0001-9654-7243, Twitter: @falconi_m

**Supplementary Materials - Index**

| **Supplementary Figures and Tables** |  |
| --- | --- |
| Figure S1  Figure S2 | *pag. 3*  *pag. 4* |
| Figure S3  Figure S4  Table S1  **References** | *pag. 5 pag. 6*  *pag. 6*  *pag. 7* |
|  |  |
|  |  |

**Supplementary Figures and Tables**

**Figure S1.**  Flowchart of patients included in the study.


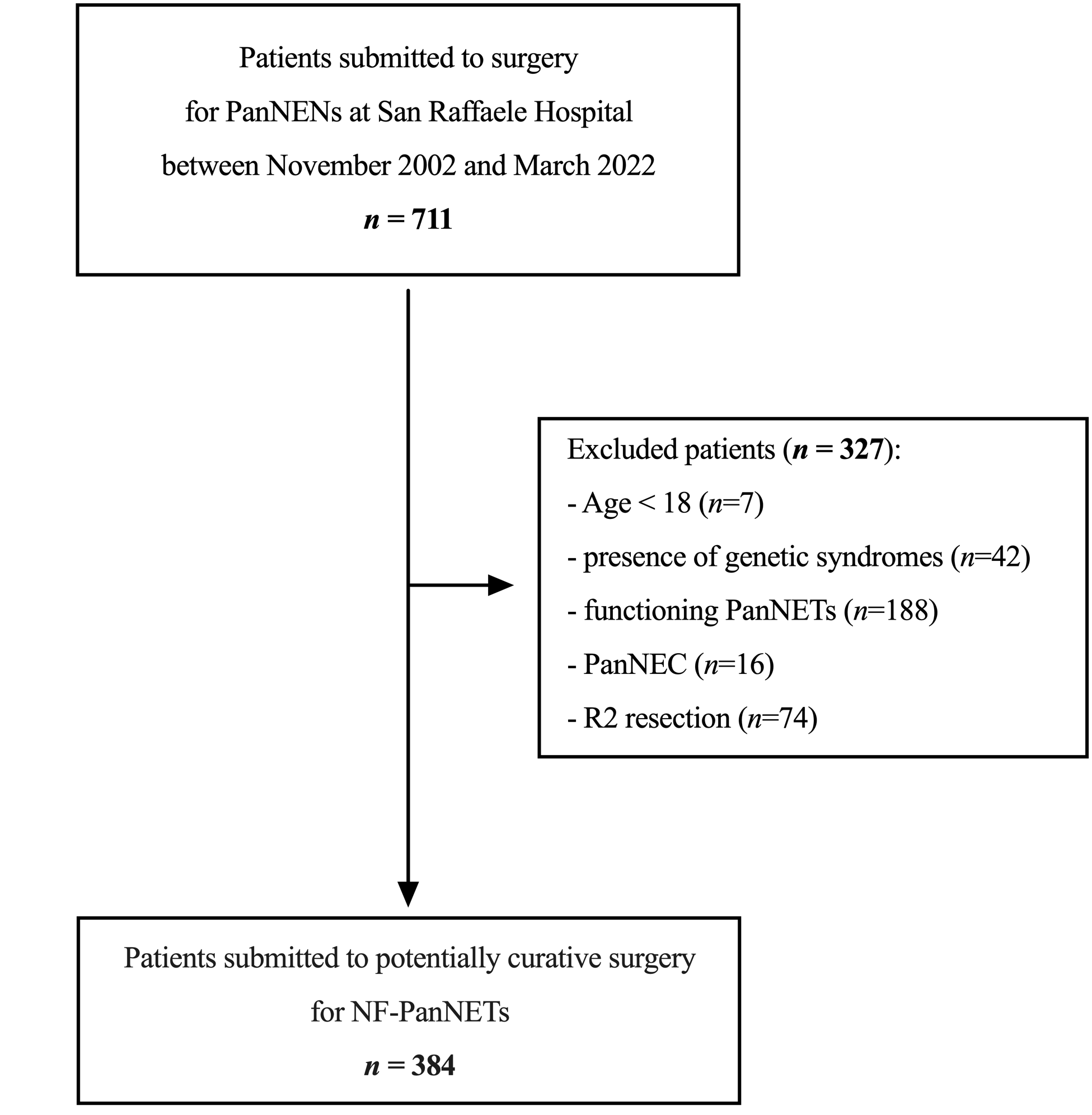


**Figure S2.** Comparison of the diagnostic workup employed in patients who underwent surgery for nonfunctioning pancreatic neuroendocrine tumors (NF-PanNETs) over the study period (2002-2022), categorized into four sub-periods.

**

**

**Figure S3.** Receiver operating characteristic (ROC) curve evaluating radiological tumour size (mm) as predictor of treatment appropriateness.

**
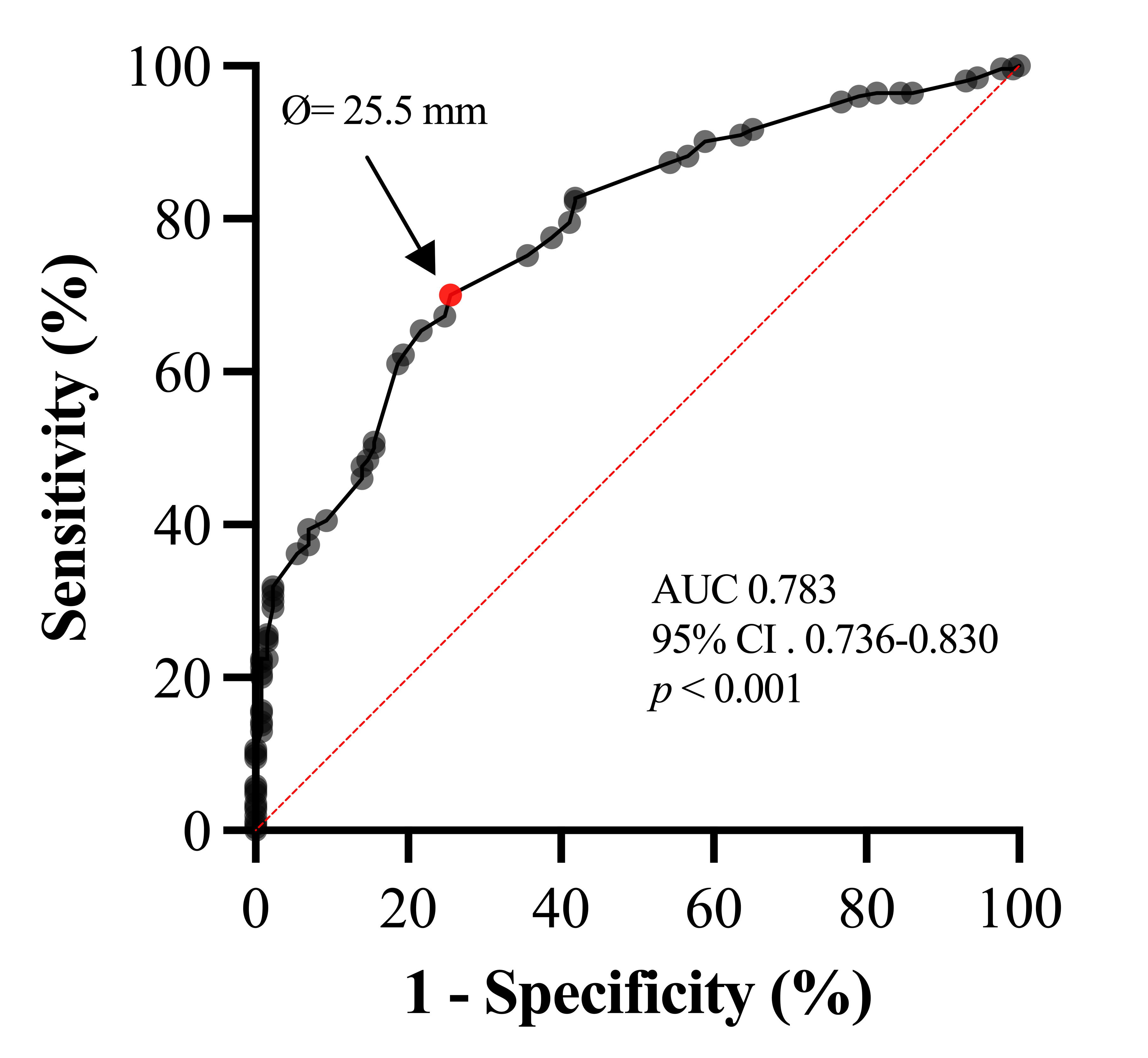
**

**Figure S4.** Comparison of (**A**) disease-free survival (DFS) and (**B**) disease-specific survival (DSS) between patients who underwent surgery for nonfunctioning pancreatic neuroendocrine tumours (NF-PanNETs) and received potential overtreatment *(n* = 129), appropriate treatment (*n* = 230) and potential undertreatment (*n* = 25).



**Table S1.** Comparison of pathological features at final histology between patients who underwent surgery for nonfunctioning pancreatic neuroendocrine tumours (NF-PanNETs) and received appropriate treatment (*n* = 230) and potential undertreatment (*n* = 25).

| Variable | Appropriate Treatment *n*=230 | Potential Undertreatment *n*=25 | *p value* |
| --- | --- | --- | --- |
| Tumor grade ^1^  G2-G3  G3 | 139 (60)  4 (2) | 20 (80)  4 (16) | 0.055  **< 0.001** |
| Ki67 index, (per cent)^*^ | 3 (2-6) | 8 (5-18) | **< 0.001** |
| T stage, T3-4 ^2^ | 81 (35) | 16 (64) | **0.005** |
| N stage, N1 ^2^ | 105 (46) | 19 (76) | **0.004** |
| M stage, M1 ^2^ | 14 (6) | 6 (24) | **0.007** |
| Microvascular invasion | 125 (54) | 22 (88) | **0.001** |
| Perineural invasion | 73 (32) | 13 (52) | **0.042** |
| Necrosis | 20 (9) | 9 (36) | **0.001** |
| Peripancreatic tissue infiltration | 43 (19) | 5 (20) | 0.793 |
| Values are *n (%)* unless otherwise indicated  * Expressed as median (i.q.r.) |  |  |  |

**References**

1 Lloyd, RV; Osamura, RY; Klopper, G; Rosai J, ed. WHO Classification of Tumours of Endocrine Organs. 4th ed. Lyon: IARC; 2017.

2 Rindi G, Kloppel G, Alhman H, et al. TNM staging of foregut (neuro)endocrine tumors: a consensus proposal including a grading system. Virchows Arch. 2006; **449**: 395–401.
