# Supplementary figures and images for: Critical appraisal of the adequacy of surgical indications for non-functioning pancreatic neuroendocrine tumours
Source: BJS Open. 2024 Aug 6;8(4):zrae083. doi: 10.1093/bjsopen/zrae083 (PMC11303005; doi:10.1093/bjsopen/zrae083)

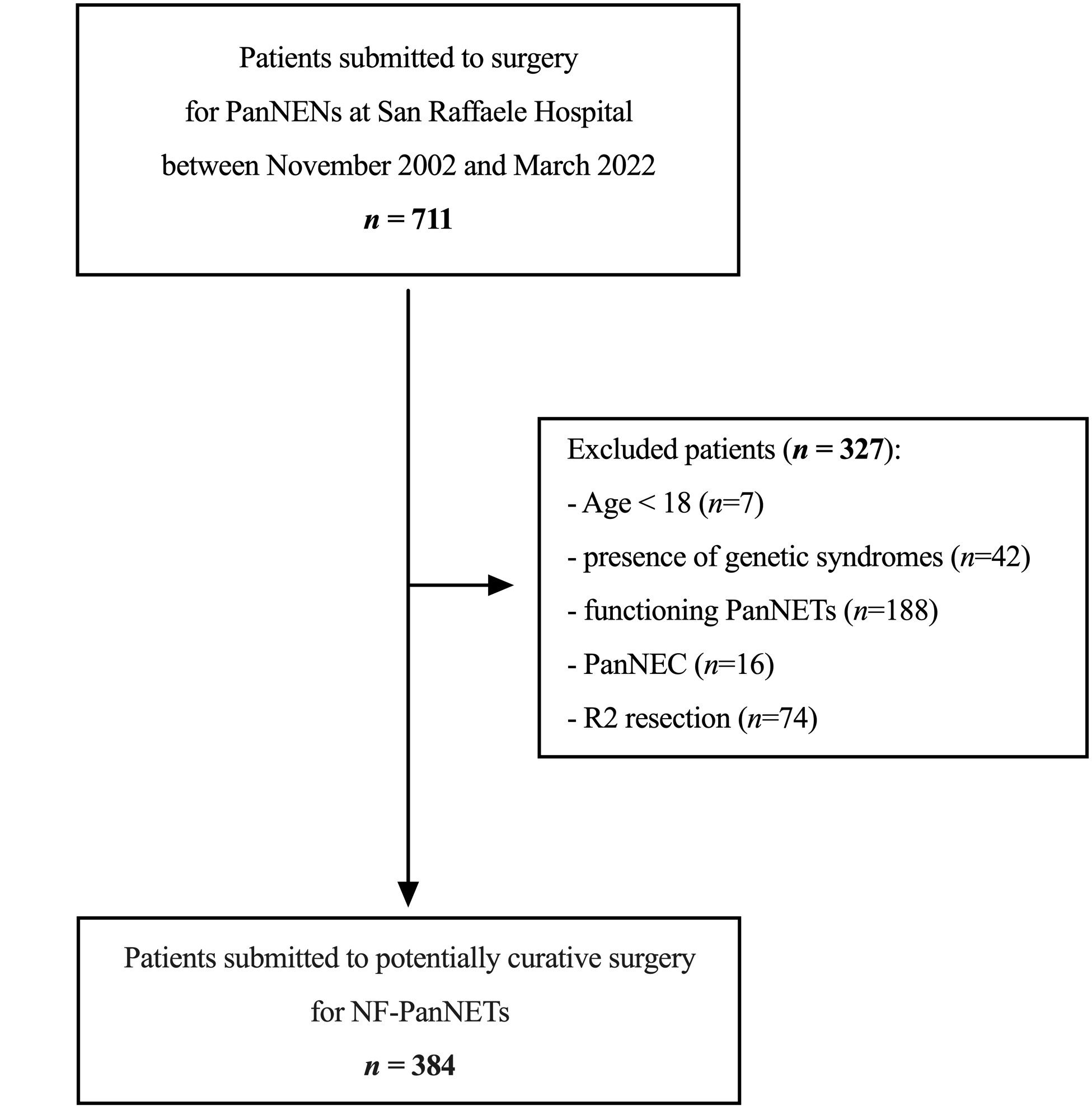

Supplement: zrae083_Supplementary_Data [file zrae083_supplementary_data.zip › Figure_S1.tiff]

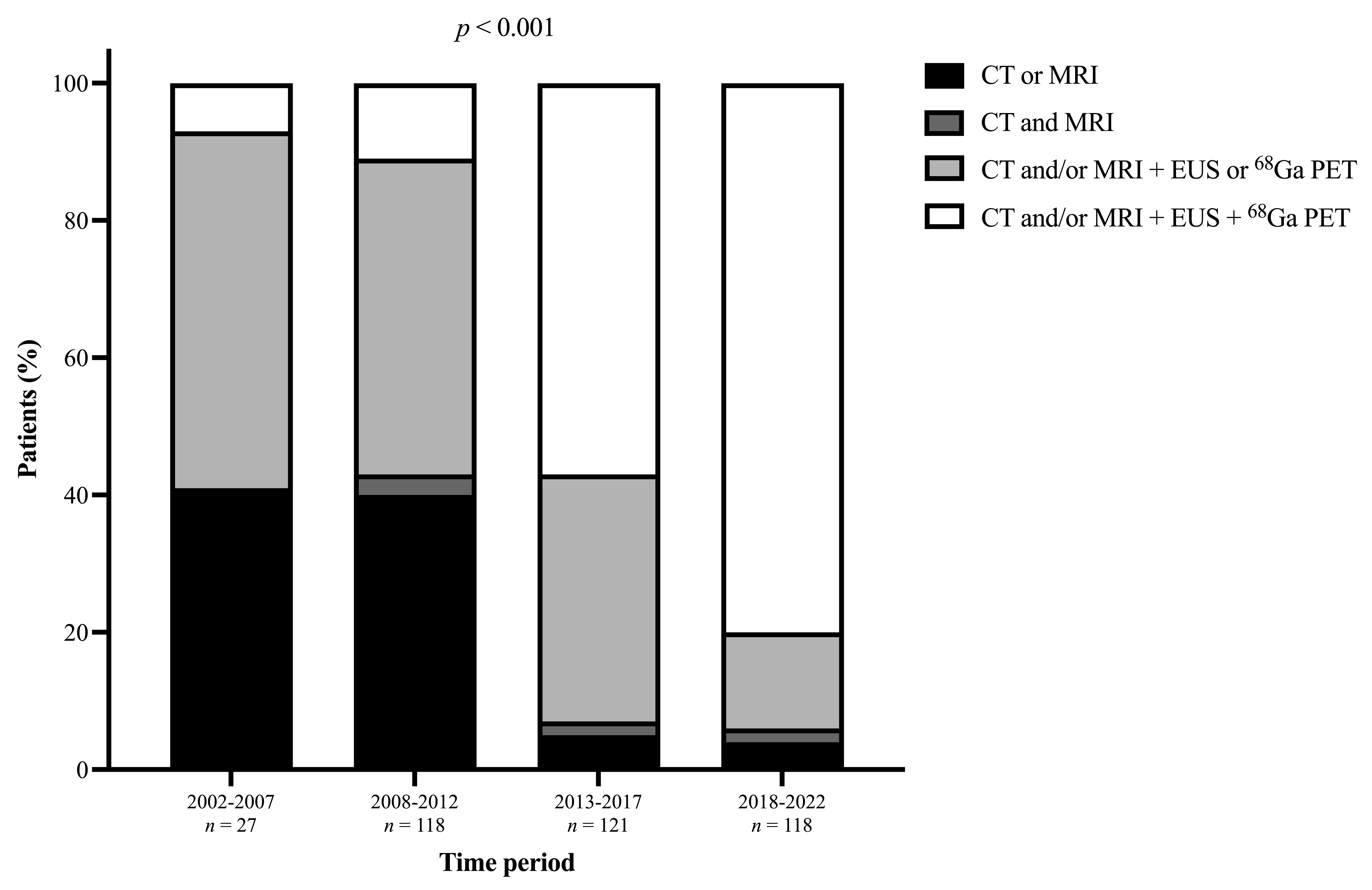

Supplement: zrae083_Supplementary_Data [file zrae083_supplementary_data.zip › Figure_S2.jpg]

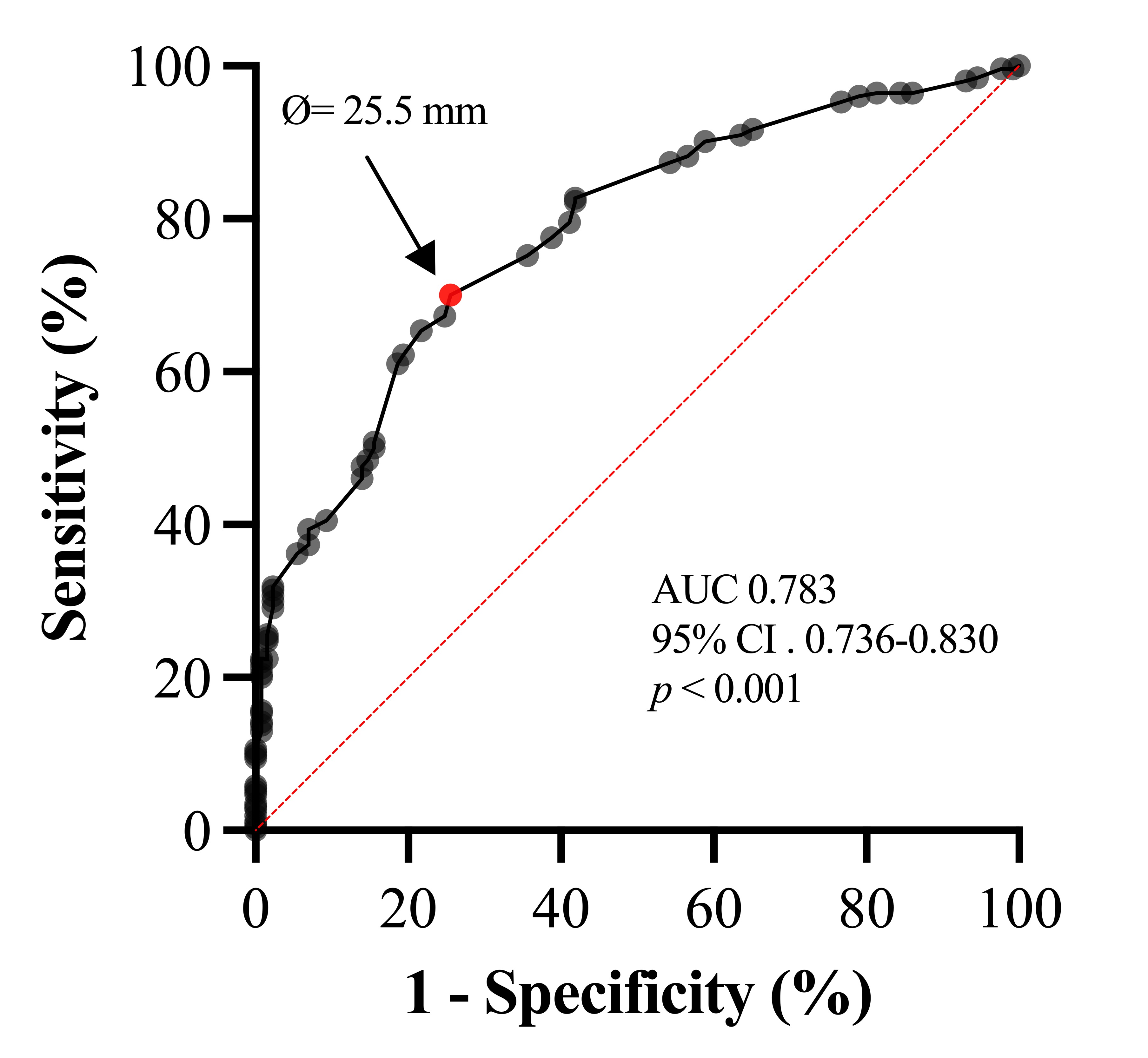

Supplement: zrae083_Supplementary_Data [file zrae083_supplementary_data.zip › Figure_S3.jpg]

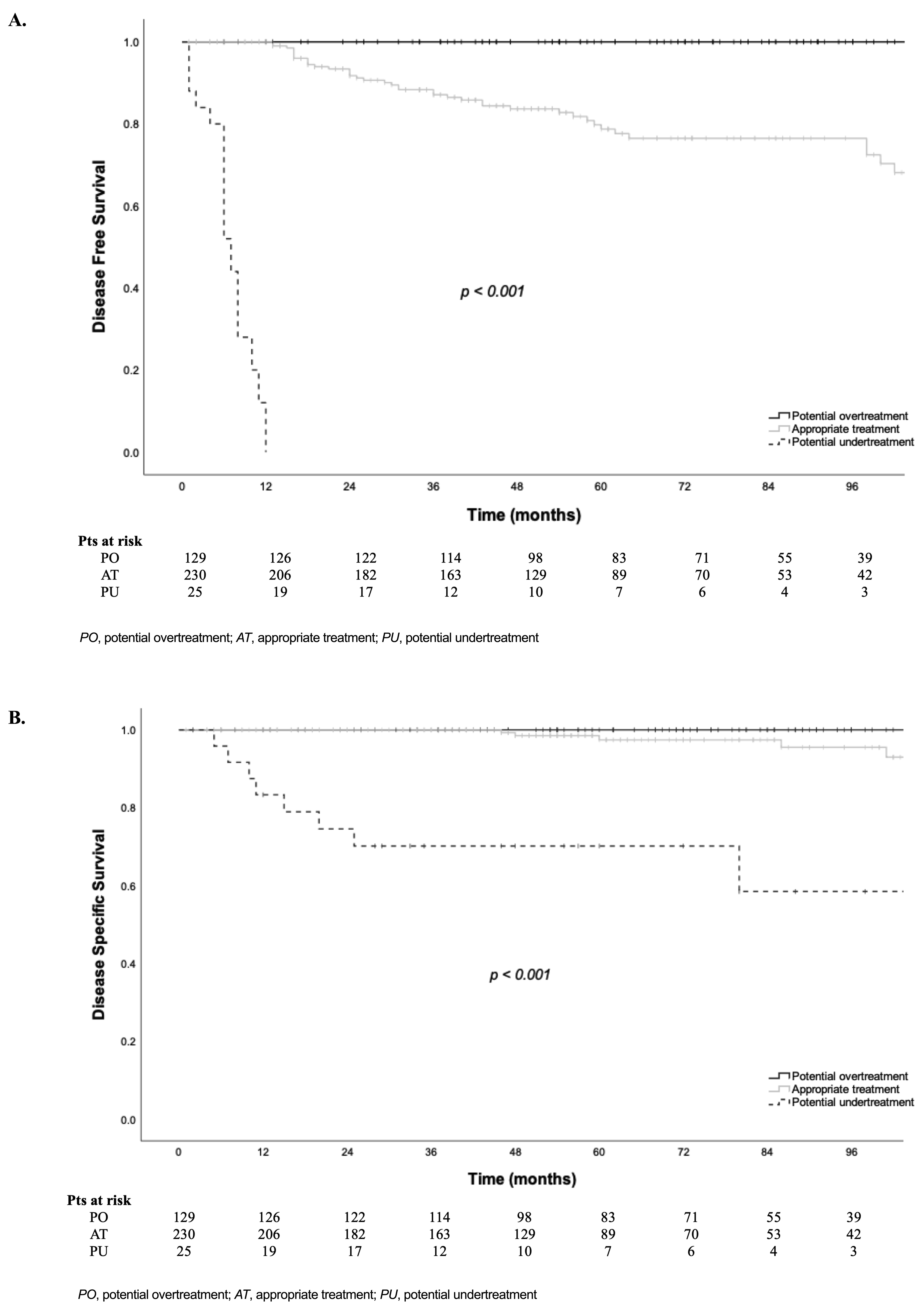

Supplement: zrae083_Supplementary_Data [file zrae083_supplementary_data.zip › Figure_S4.jpg]
